# Genetic and Clinical Characteristics of Central Serous Chorioretinopathy With Steroid Use

**DOI:** 10.7759/cureus.58631

**Published:** 2024-04-20

**Authors:** Seigo Yoneyama, Ayumi Fukui, Yoichi Sakurada, Nobuhiro Terao, Natsuki Kusada, Wataru Kikushima, Yumi Kotoda, Fumihiko Mabuchi, Chie Sotozono, Kenji Kashiwagi

**Affiliations:** 1 Department of Ophthalmology, University of Yamanashi, Yamanashi, JPN; 2 Department of Ophthalmology, Kyoto Prefectural University of Medicine, Kyoto, JPN

**Keywords:** clinical characteristics, complement factor h, gender, steroid use, central serous chorioretinopathy

## Abstract

Purpose: To compare the genetic and clinical characteristics of central serous chorioretinopathy (CSC) in patients with and without steroid use.

Methods: A total of 407 consecutive patients with CSC were included. Demographic data and clinical factors, including subfoveal choroidal thickness, bilateral involvement, descending tracts, pachydrusen, fibrin, and dome-shaped pigment epithelial detachment, were obtained. Variants of complement factor H (CFH)* *I62V (rs800292) and rs1329428 were genotyped in all cases using TaqMan technology.

Results: Of the total patients, 48 (11.8%) were steroid users. The majority of males were non-steroid users (82.5%) than steroid users (58.3%) (p = 9.8 × 10^-5^). Demographic data and the prevalence of clinical factors were comparable between the two groups (all p-values > 0.10). Risk allele frequencies of CFH rs800292 and rs1329428 were also comparable between the two groups (p = 0.76, rs800292: steroid users = 52.1% vs. non-steroid users = 50.4%; p = 0.62, rs1329428: steroid users = 47.9% vs. non-steroid users = 45.3%).

Conclusions: Except for the male/female ratio, there were no significant differences in the clinical presentation or genetic characteristics, including variants of the CFH gene, between the two groups.

## Introduction

Central serous chorioretinopathy (CSC) mainly affects middle-aged men and is characterized by concentric serous retinal detachment of the macula [1,2]. Although the etiology of CSC has not been completely elucidated, choroidal circulatory disturbances are thought to be a major risk factor, along with vulnerability of the retinal pigment epithelium (RPE) and scleral thickness. Furthermore, male sex, stress, testosterone, exposure to exogenous corticosteroids, and pregnancy are involved in the development of CSC [1,3-6].

Although some studies have reported that corticosteroid use is a risk factor for the development of CSC [3,4,6], it is unclear why corticosteroid use is a risk factor for developing CSC. Many studies have reported that the choroid in eyes with CSC is thicker than that in normal eyes [7,8]. A thick choroid is considered a risk factor for CSC development, and several studies have compared choroidal thickness between steroid-induced CSC eyes and idiopathic CSC eyes. However, there is no consensus on choroidal thickness in patients with steroid-induced CSC. For example, Honda et al. reported that the choroidal thickness in steroid-induced CSC patients was thinner than or similar to that in idiopathic CSC patients [9]. Conversely, Araki et al. reported that the choroidal thickness in steroid-induced CSC patients was thicker than that in idiopathic CSC patients [10]. Some studies have reported that corticosteroids suppress the repair mechanism of the RPE and may affect the choroidal circulation and choroidal barrier to induce CSC. However, its exact mechanism of action remains unclear.

Several candidate gene approaches and genome-wide association studies have been performed for CSC, and the complement factor H (CFH) gene is associated with CSC in different ethnicities [11-13]. However, few studies have compared the association between CFH variants and steroid-induced CSC.

The pathogenesis of steroid-induced CSC and idiopathic CSC may be different, and the clinical findings of CSC may also differ. In this study, we investigated the differences in clinical findings and genetic characteristics of CSC patients depending on their history of steroid use.

## Materials and methods

Subjects

A retrospective medical chart review was performed for 407 consecutive patients diagnosed with CSC between April 2016 and March 2023 at the Macular Clinic, Department of Ophthalmology, University of Yamanashi Hospital and the Kyoto Prefectural University of Medicine Hospital. CSC was diagnosed as an eye with subretinal fluid in the macular region on optical coherence tomography (OCT) together with leakage on fluorescein angiography (FA) and choroidal vascular hyperpermeability on indocyanine green angiography (ICGA). The method of steroid administration was investigated through interviews. During the study period, all eyes diagnosed as CSC were enrolled in the present study. Eyes with exudative age-related macular degeneration, geographic atrophy, and other macular abnormalities, including angioid streaks, myopic choroidal neovascularization, and other secondary choroidal neovascularization, were excluded.

All patients underwent comprehensive ophthalmologic examination, which included measurement of best-corrected visual acuity (BCVA), dilated fundus evaluation with 78 D condensing lens, color fundus photography, fundus autofluorescence (FAF) using the Optos California (Optomap, Optos plc, Dunfermline, Scotland, UK), FA/ICGA using a confocal scanning laser ophthalmoscopy (HRA2, Heidelberg Engineering Inc, Dossenheim, Germany), and OCT using swept-source OCT (Atlantis/Triton, Topcon, Japan) or spectral domain OCT (Spectralis HRA2, Heidelberg Engineering Inc, Dossenheim, Germany).

Subfoveal choroidal thickness (SCT) was measured as the vertical distance between the outer border of the RPE and the choroidoscleral border using OCT images. The descending tract was a downward-leading swathe with decreased autofluorescence originating from the posterior pole and extending below the level of the inferior arcade. Pachydrusen was diagnosed if the following three criteria were included: (1) drusen diameter was > 125 μm; (2) the outer contours of the drusen were irregular; (3) drusen occurred in isolation or in groups of only a few drusen, showing yellowish scattered deposits over the posterior pole. Late-phase ICGA was used to differentiate pachydrusen, which exhibited punctate hyperfluorescent spots, from soft drusen, which showed hypofluorescence. Fibrin was defined as a round white lesion and subretinal hyperreflective material above the RPE on OCT. Dome-shaped pigment epithelial detachment (PED) was defined as a PED width larger than 500 µm. If both eyes were affected, values for the last affected eye were used. If the last affected eye was unknown, the values for the right eye were adopted in the study. The presence or absence of clinical characteristics of CSC was independently evaluated based on multimodal imaging by four retinal specialists (Y.S. or S.Y/N.T. or A.F.).

This retrospective study was reviewed and approved by the Ethics and Gene Analysis Committee in the Faculty of Medicine, University of Yamanashi and Kyoto Prefectural University of Medicine. This study adhered to the principles of the Declaration of Helsinki.

Genotyping

Genotyping was performed for all patients. Peripheral blood samples (5 ml) were extracted at baseline FA/ICGA. The genomic DNA was purified using a PUREGENE DNA Isolation Kit (Gentra Systems, Minneapolis, MN). We genotyped CFH rs800292 and rs1329428 in the present study because these variants have been reported to be strongly associated with CSC in the Japanese population. Genotyping was performed in all cases using TaqMan genotyping assays on a 7500 Real-Time PCR System (Applied Biosystems, Foster City, CA) in accordance with the manufacturer’s recommendations.

Statistical analysis

BCVA measured using a Landolt chart was converted to the logarithm of the minimal angle of resolution (logMAR) for statistical analysis. Statistical analyses were performed using IBM SPSS Statistics version 28 for Windows (IBM Corp., Armonk, NY). Differences in categorical variables between groups were tested using the chi-square test. Differences in continuous variables were tested using the Mann-Whitney U test. Statistical significance was set at p < 0.05.

## Results

A total of 407 patients with CSC were included in this study. Table 1 shows the clinical and genetic characteristics of the patients with CSC at initial presentation (male/female: 324/83; mean age: 55.9 ± 12.1 years). Among all the patients, 48 (11.8%) had a history of steroid use. Table 2 shows a comparison of the clinical and genetic characteristics between patients with and without steroid use. The average SCT was 388 ± 114 µm and 376 ± 100 µm in non-steroid and steroid users, respectively. Patients without steroid use demonstrated a significantly higher proportion of males than those with steroid use (82.5% vs. 58.3%, p = 9.8 × 10 - 5). There were no significant differences in age, SCT, baseline BCVA, bilateral involvement, or clinical findings between patients with and without steroid use. The risk allele frequencies of CFH, including those of rs800292 and rs1329428, were not significantly different between patients with and without steroid use. Table 3 shows the causal diseases among the steroid users. The most common diseases caused by steroid use were collagen (29.1%) and skin diseases (25.1%). The methods used for steroid administration are listed in Table 4. Systemic administration is the most commonly used method of treatment. As most steroids are administered internally or intravenously to treat collagen diseases, systemic administration was the most common method of administration.

**Table 1:** Baseline characteristics of patients with central serous chorioretinopathy BCVA: best-corrected visual acuity; logMAR: logarithm of the minimal angle of resolution; CFH: complement factor H.

Gender, male	324 (79.6%)
Age (years)	55.9 ± 12.1
Steroid use	48 (11.8％)
Bilateral involvement	110 (27.0%)
Subfoveal choroidal thickness (μm)	387 ± 113
Baseline BCVA (logMAR)	0.17 ± 0.33
Descending tract(s)	52 (12.8%)
Pachydrusen	103 (25.3%)
Fibrin	34 (8.4%)
Dome-shaped pigment epithelial detachment	58 (14.3%)
CFH I62V (rs800292)	
GG	99
GA	204
AA	104
A allele frequency	50.6%
CFH (rs1329428)	
CC	115
CT	213
TT	79
T allele frequency	45.6%

**Table 2:** Comparison of characteristics between non-steroid users and steroid users BCVA: best-corrected visual acuity; logMAR: logarithm of the minimal angle of resolution; CFH: complement factor H.

	Without steroid use (n = 359)	With steroid use (n = 48)	p-value
Gender, male	296 (82.5%)	28 (58.3%)	9.8 × 10^-5^
Age (years)	56.1 ± 12.0	54.8 ± 12.7	0.40
Bilateral involvement	96 (26.7%)	14 (29.2%)	0.72
Subfoveal choroidal thickness (μm)	388 ± 114	376 ± 100	0.40
Baseline BCVA (logMAR)	0.18 ± 0.33	0.11 ± 0.32	0.87
Descending tract(s)	48 (13.4%)	4 (8.3%)	0.33
Pachydrusen	94 (26.2%)	9 (18.8%)	0.27
Fibrin	27 (7.5%)	7 (14.6%)	0.10
Dome-shaped pigment epithelial detachment	50 (13.9%)	8 (16.7%)	0.61
CFH I62V (rs800292)			
GG	89	10	0.76
GA	178	26	
AA	92	12	
A allele frequency	50.4%	52.1%	
CFH (rs1329428)			
CC	102	13	0.62
CT	189	24	
TT	68	11	
T allele frequency	45.3%	47.9%	

**Table 3:** Diseases that led to steroid use

	Total number (n = 48)
Collagen diseases	14 (29.1％)
Skin diseases	12 (25％)
Asthma	8 (16.7%)
Kidney diseases	3 (6.3％)
Neurological diseases	2 (4.2％)
Other diseases	9 (18.7％)

**Table 4:** Methods of steroid administration

	Total number (n = 48)
Systemic administration	29 (60.4％)
Transdermal administration	10 (20.8％)
Inhalation	7 (14.6%)
Subtenon injection	1 (2.1％)
Intranasal administration	1 (2.1％)

## Discussion

A previous genome-wide association study demonstrated that variants of CFH I62V and rs1329428 were associated with SCT in normal participants and CSC development [13]. In this study, the risk allele frequency of the CFH gene was almost the same between the two groups, and SCT was not significantly different between the two groups. To date, no studies have investigated the genetic background of steroid-induced CSC; however, it may have the same genetic background in terms of variants of the CFH gene as idiopathic CSC. In addition to CFH variants, age, and axial length are reportedly associated with choroidal thickness [14,15]. Although axial length was not measured in all patients in this study, age was also almost the same between the two groups, which explains why the SCT between these two groups was not significantly different. The prevalence of clinical findings was not significantly different between the two groups. The mean age between the two groups was not significantly different. As pachydrusen is associated with older age [16], the prevalence of pachydrusen might be almost the same between the two groups. We recently reported that RPE alterations greater than 2-disc areas, including descending tract(s) and patchy atrophy, are exclusively seen in males [17]. In the present study, the prevalence of descending tracts was not significantly different between the two groups, although the prevalence in males was significantly higher in non-steroid users than in steroid users. Considering male patients exclusively, the prevalence of the descending tract was 16.2% (48/296) and 14.3% (4/28) in non-steroid and steroid users, respectively, suggesting that the prevalence was almost the same between the two entities.

The prevalence of female sex was significantly higher in steroid users than in non-steroid users. This is because collagen and skin diseases are more prevalent in women than idiopathic CSC [18,19].

This study has some limitations. Because the proportion of steroid users was small, the number of patients with steroid-induced CSC was limited. To confirm or refute this tentative conclusion, it is necessary to include more patients who use steroids.

## Conclusions

We compared the genetic and clinical characteristics of CSC in patients with and without steroid use. Steroid use was observed in 11.8% of the patients with CSC. The prevalence of female sex was significantly higher in steroid users than in non-steroid users. Except for the male/female ratio, there were no significant differences in the clinical presentation and genetic characteristics of CSC patients depending on their history of steroid use. In CSC, steroid use may not influence the clinical presentation or genetic background.
